# Perihippocampal failure after hippocampal-avoidance brain radiotherapy in small cell lung cancer patients: Cases report and literature review

**DOI:** 10.1097/MD.0000000000038884

**Published:** 2024-07-12

**Authors:** Yi-Chia Ho, Li-Tsun Shieh, Chia-Hui Lin, Chia-Chun Chen, Sheng-Yow Ho

**Affiliations:** a Department of Internal Medicine, National Taiwan University Hospital, Hsin-Chu Branch, Hsin-Chu, Taiwan; b Department of Radiation Oncology, Chi Mei Medical Center, Liouying, Tainan, Taiwan; c Department of Radiation Oncology, Chi Mei Medical Center, Tainan, Taiwan.

**Keywords:** brain metastasis, hippocampal-avoidance, perihippocampal failure, small cell lung cancer, whole-brain radiotherapy

## Abstract

**Rationale:**

Brain metastasis is a major concern, and may occur in roughly 50% of patients during the clinical course of small cell lung cancer (SCLC). Because prophylactic cranial irradiation reduces the incidence of brain metastases and improves overall survival, prophylactic cranial irradiation is recommended for SCLC patients without distant metastases or an extensive stage and have responded well to systemic therapy. Hippocampal-avoidance whole-brain radiotherapy (HA-WBRT) is preferred to preserve hippocampal function while minimizing negative cognitive effects.

**Patient concerns:**

Reducing the dose delivered to the hippocampus below the therapeutic brain dose may increase the risk of hippocampal progression; thus, HA-WBRT may be associated with a risk of perihippocampal recurrence.

**Diagnosis:**

Three patients with SCLC received HA-WBRT and developed intracranial failure during clinical follow-up; 3 relapsed with intracranial failure in the perihippocampal region after 12, 13, and 7 months, respectively.

**Intervention and outcomes:**

Compared to the therapeutic brain dose of cases and the underdose region around the HA region, we matched MRI scans of intracranial failure and previous planning scans of simulation and found a deviation of the underdosed region within the perihippocampal failure of approximately 55% to 63%.

**Lessons:**

Perihippocampal failure is a rare clinical outcome in SCLC patients following HA-WBRT. Perihippocampal failure could be caused by an underdose of radiation or by the aggressiveness of the cancer itself. More research into this topic is encouraged.

## 1. Introduction

Small cell lung cancer (SCLC) is the most aggressive disease, with poor survival rates. Although most patients respond to initial therapy, the disease usually progresses rapidly within months. Brain relapses are a major concern, affecting approximately 50% of patients during the clinical course of SCLC. Because prophylactic cranial irradiation (PCI) reduces the incidence of brain metastases and improves overall survival, PCI is recommended for SCLC patients without distant metastases or an extensive stage and have responded well to systemic therapy.^[1–6]^ Conventional whole-brain radiotherapy (WBRT) was previously the standard treatment for SCLC patients, but there are concerns that cranial irradiation may cause neurocognitive decline due to radiation-induced injury to healthy brain tissue. Hippocampal neural stem cell injury following WBRT may play an important role in neurocognitive function, particularly in learning and memory.^[1–3]^ Hippocampal-avoidance (HA)-WBRT, which has evolved from modern radiotherapy techniques, allows for the treatment of the entire brain while limiting the dose to both hippocampi, potentially preserving hippocampal function. Recent clinical research recommends that hippocampus sparing could be a viable and dependable technique for reducing adverse cognitive effects in WBRT.^[1–3,7–11]^

Perihippocampal failure is a rare clinical complication in patients with SCLC after HA-WBRT. The risk of perihippocampal failure following HA-WBRT is unknown in SCLC patients, but it may be theoretically higher than the counterpart of non-small-cell lung cancer.^[8,11–13]^ Its clinical features have yet to be fully elucidated due to a lack of clinical data; thus, clinical cases must be observed. Herein, we present 3 cases of developing perihippocampus failure following HA-WBRT in SCLC patients and evaluate the relationship between the underdose of therapeutic cranial radiation and brain failure in the HA region.

## 2. Cases presentation

### 2.1. Case 1

**Figure 1.**
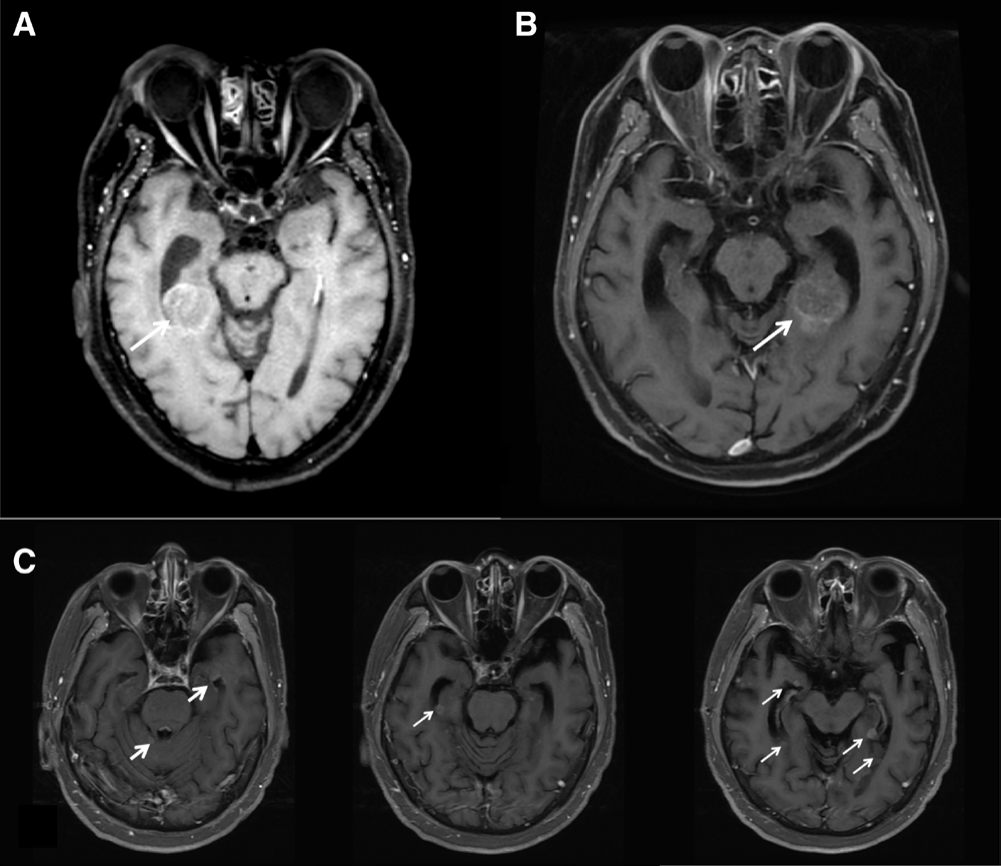
Intracranial perihippocampal failure in 3 small cell lung patients after hippocampal-avoidance whole-brain radiotherapy. (A) Patient A developed a single intracranial failure in the right perihippocampus region 12 months after HA-PCI. (B) Patient B discovered perihippocampal failure around the left hippocampus 13 months after HA-PCI. (C) Patient C reported multiple intracranial recurrences around the bilateral hippocampi, the temporal horn of the lateral ventricle, and quadrigeminal cistern 6 months after HA-whole-brain radiotherapy (arrows in A–C). HA-PCI = hippocampal-avoidance prophylactic cranial irradiation.

The first case involved a 64-year-old man with clinical presentation of a right neck mass and coughed for several months, prompting his primary care physician to evaluate his lung condition in June 2020. Chest computed tomography (CT) revealed a right lung tumor, 57 mm in size, with invasion of the right hilum, right main bronchus, and enlarged lymphadenopathy in the mediastinum and right low neck region. Bronchoscopy revealed tumorous lesions along the right main bronchus, extending to segments of the right upper lobe, and a tumor biopsy confirmed SCLC. Following the stage work-up, the clinical stage was assigned to a limited-stage, or T4N3M0 stage IIIC, using the 8th edition staging system of the American Joint Committee on Cancer. He was a heavy smoker with 2 packs per day for over 30 years and suffered from medical diseases including chronic obstructive pulmonary disease, hypertension, and diabetes mellitus. He underwent 2 cycles of chemotherapy with the regimen of cisplatin and etoposide, followed by concurrent chemoradiotherapy to treat lung tumor and gross mediastinal lymphadenopathy (66 Gy in 33 fractions) from July to September 2020. Follow-up chest CT revealed significant regression of the lung tumor; thus, HA-PCI had prescribed with the dose of 25 Gy in 10 fractions in October 2020. Due to concerns about the early development of recurrence, the patient signed informed consent after a thorough discussion with the oncologist and enrolled in the clinical trial with tremelimumab (cytotoxic T lymphocyte-associated antigen-4 blocking antibody) and durvalumab since November 2020. Until October 2021, the patient complained of headaches, and brain magnetic resonance imaging (MRI) revealed a single metastatic lesion (43 × 23 × 23 mm) in the right hippocampus region, with compression to the right lateral ventricle (Fig. 1A). Stereotactic radiosurgery was done to treat right hippocampal brain metastases (35 Gy in 5 fractions) using the HyperArc™ technique in November 2021. The disease had progressed, and a follow-up brain MRI revealed regression of the right hippocampus tumor as well as progression to multiple brain metastases. The patient died as a result of deteriorating brain symptoms at the end of May 2022.

### 2.2. Case 2

A 62-year-old male was admitted to the ward in August 2017 after complaining of a worsening cough and copious sputum for several months. He was a current smoker with a 30-year smoking history, as well as an oncologic history of left renal cell carcinoma after following nephrectomy in another hospital 13 years ago, with no clinical signs of disease. He took medications to treat hypertension, diabetes mellitus, and hyperuricemia. Chest CT revealed a large left lower lung tumor, 80 mm in size, with enlarged lymph nodes in the mediastinum. Endobronchial ultrasound-guided biopsy revealed SCLC. The image stage work-up revealed no obvious lung, visceral, or brain metastasis. The clinical stage was T4N2M0 (stage IIIB), also known as the limited-stage. Two cycles of systemic chemotherapy with carboplatin and etoposide regimen were administered first, followed by concurrent chemoradiotherapy with a 60-Gy radiation dose delivered in 30 fractions from October to November 2017. A follow-up chest CT revealed nearly complete regression of the left lung tumor, leaving only interstitial pulmonary fibrosis. Therefore, HA-PCI was prescribed in a dose of 27 Gy divided into 15 fractions in January 2018. He had repeated hemoptysis from April to December 2018, and repeated bronchoscopy revealed only narrowing of the bronchus and the presence of blood clots, but no tumor recurrence. He experienced unsteady gait and altered consciousness, and a brain MRI revealed a recurrent tumor in the left hippocampus region (Fig. 1B) in February 2019. Salvage focal radiotherapy of 30 Gy in 10 fractions was administered to the hippocampus lesion, but his condition deteriorated and he quickly succumbed to the disease.

### 2.3. Case 3

A 65-year-old male was admitted to the hospital due to a cough with sputum that persisted for several months in June 2022. The patient was a current smoker with a 40-year smoking history and had chronic obstructive pulmonary disease. Chest CT revealed a 90-mm left lung mass that attaches to the heart, as well as the presence of growing lymph nodes in the mediastinum. Endobronchial echo-guided biopsy revealed SCLC. Brain metastasis was not discovered during the stage work-up. The clinical stage was T4N2M0, stage IIIB, or limited-stage. During the period from July 2022 to January 2023, 6 cycles of systemic chemotherapy with the regimen of cisplatin and etoposide were administered, followed by concurrent chemoradiotherapy with a total of 60 Gy delivered in 30 fractions to treat the involved lung tumor. Only a partial response was observed in the follow-up image study, so second-line chemotherapy was supplemented with a topotecan regimen in the first 3 months of 2023. The clinical restage revealed a partial response to the lung tumor. However, he had no neurological brain symptoms, and a follow-up brain MRI accidentally revealed only 2 small brain metastases that were not within 5 mm of the bilateral hippocampi. He received brain radiation (30 Gy in 10 fractions) using the HA-WBRT technique in June 2023. Unfortunately, he developed headache, unsteady gait, and double vision in December 2023, and a brain MRI revealed brain metastases around the bilateral hippocampus, which was medial to the temporal horn of the lateral ventricle and lateral to the quadrigeminal cistern (Fig. 1C). Salvage focal irradiation was performed on each of the brain metastases with a dose of 40 Gy in 10 fractions. The neurological symptoms progressed to low-limb paraplegia, and a spine MRI revealed metastatic spine lesions involving the 10th thoracic vertebral body and spinal cord invasion. His condition deteriorated, and he succumbed to the disease quickly.

## 3. Dosimetric analysis of hippocampal failure

**Figure 2.**
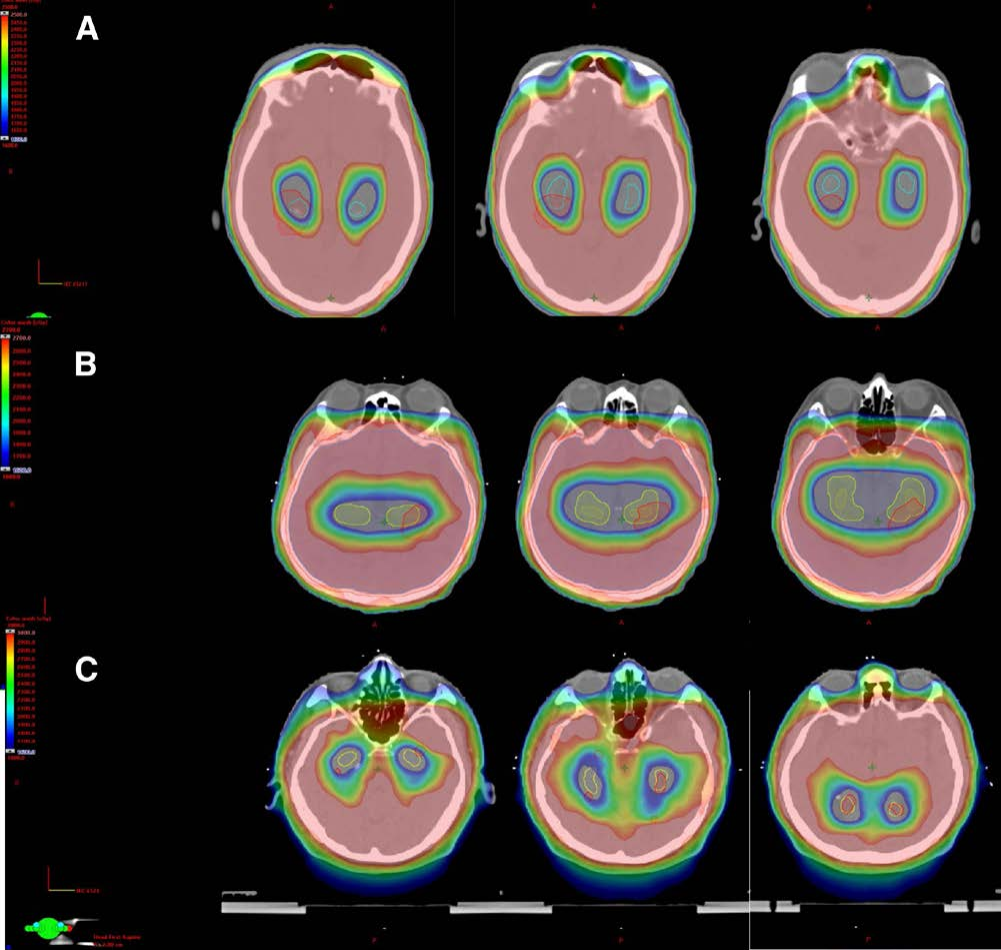
MRI of patients (A–C) with intracranial failure with matched follow-up MRI scans and planning CT-simulation scans around the underdose distribution map for hippocampal-avoidance cranial irradiation. These registration images demonstrate that the region avoiding the hippocampus coincides with the occurrence of perihippocampal failure. The color wash dose ranges from 16 Gy to the prescribed amount of radiation (25–30 Gy).

Hippocampal contouring and HA-WBRT planning directives of 3 cases were guided by the RTOG 0933 criteria,^[10]^ and the technique and clinical study are described in our previous study.^[14,15]^ To investigate whether a deviation of underdose in the HA region led to perihippocampal failure, we assessed the parameters of dosimetry in the HA-WBRT planning of our cases (Table 1). We compared the brain dose of patients A to C and the underdose region around the HA region by maping the MRI scans of intracranial failure and previous planning CT scan of simulation and found that a deviation of the underdosed region within the perihippocampal failure was approximately 55% to 63%. Figure 2 depicts fusion MRI images of perihippocampal failure with the previously planned dose distribution map of HA-WBRT.

**Table 1:** Dosimetric analysis of perihippocampal failure after hippocampal-avoidance whole-brain radiotherapy in patients with small cell lung cancer.

Characteristics	Case A	Case B	Case C
Purpose of radiation	PCI	PCI	Palliation
Hippocampus volume, mL	2.8	3.5	7.0
HA volume, mL	26.0	30.8	51.0
Brain volume, mL	1346	1598	1339
HA volume/brain volume (%)	1.9	1.9	3.8
Prescribed dose (Gy)	25	27	30
Perihippocampal failure volume, mL	21.1	17.1	10.2
Medium dose within the hippocampal failure, (range) (Gy)	23.5 (8.6–27.3)	16.9 (7.1–30.9)	14.3 (8.9–27.3)
Under-dose within the hippocampal failure (%)	63.0	55.8	54.6

HA = hippocampal-avoidance; PCI = prophylactic cranial irradiation.

## 4. Discussion

The hippocampus is a curved structure deep in the temporal lobe that is adjacent to the lateral ventricles of the brain. It is involved in numerous cognitive processes, including learning, memory, and emotion. Clinical studies have shown that even low doses of radiation cause an early and significant decline in neurogenesis in the affected hippocampus.^[1]^ Prospective phase II and randomized phase III SCLC trials have all confirmed the efficacy of HA-WBRT in preserving neurocognitive function,^[2,3]^ though a different randomized trial found no decline in cognition in SCLC patients receiving HA-PCI compared to conventional PCI.^[4]^ The use of the HA-WBRT technique may be recommended as the standard of care for limited-stage or countable brain metastatic lesions without metastasis around the HA region and good performance status in SCLC patients^[5,8]^ The newly revised guidelines for limited-stage SCLC, HA-WBRT is recommended as a new strategy of radiation therapy for preserving cognition and memory.^[5]^

Reducing the dose delivered to the HA region below the therapeutic level may increase the risk of hippocampal progression; thus, HA-WBRT may be associated with a risk of hippocampal recurrence. Previous randomized studies have found low rates of perihippocampal metastases ranging from 1% to 8.8% incidence in cancer patients with brain metastasis following HA-WBRT.^[9,10]^ Clinical decision-makers should balance the risk of perihippocampal failure against the preservation of neurocognitive function. As we know, SCLC patients can have a higher incidence of brain metastases of more than 50% depending on the stage of the disease, so it is assumed that the entire brain is seeded with micrometastatic disease, even if no intracranial lesions are detected in imaging studies.^[8]^ Furthermore, Korkmaz Kirakli and Oztekin^[11]^ examined the cranial MRI of 54 SCLC patients and discovered that the hippocampal metastasis estimated rate was 32% (17 patients), with a total of 4.4% of all metastases involved the HA area. However, in the real world, a randomized phase III GICOR-GOECP-SEOR study reported that only-one SCLC patient developed isolated brain failure in the hippocampal region after HA-PCI during a median follow-up time of 40.4 months,^[3]^ and another randomized phase III trial found no patient developing a metastasis within the hippocampus or underdosed region during a median follow-up of 26.6 months.^[4]^ However, the risk of developing perihippocampal failure following HA-PCI or HA-WBRT is unknown. We conducted a literature review to determine the potential incidence of perihippocampal failure following HA-PCI in the databases of MEDLINE and PubMed. Table 2 contains reports from various clinical case studies or clinical trials.^[2–4,12,16]^ According to the findings, the incidence of brain failure ranged from 6.8% to 20%, while perihippocampal failure following HA-PCI ranged from 0% to 10%. Additional evidence of this phenomenon will be provided by clinical observations and further trials. Patients A and B, who had limited disease, developed a single cranial failure in the perihippocampal region after HA-PCI 12 and 13 months, respectively. A similar case report described a patient with limited-stage SCLC who underwent HA-PCI and then developed a single brain metastasis in the HA region 7 months later.^[17]^ Patient C progressed into multiple perihippocampal failure and leptomeningeal carcinomatosis 6 months after HA-WBRT for metastatic brain disease, and we also found a similar case report in the literature.^[18]^

**Table 2:** The incidence of developing brain or perihippocampal failure after hippocampal-avoidance cranial irradiation in small cell lung cancer study.

Study (published year) [reference]	Type of study	Purpose of study	Enrolled patients	Brain failure (n, %)	Perihippocampal failure (n, %)	Median follow-up time (month)
Redmond et al (2017)^[16]^	Single center	NCF in HA-PCI	20	4 (20)	2 (10)	16.7
SAKK 1512 study (2020)^[2]^	Multi-center phase 2	NCF in HA-PCI	44	3 (6.8)	0 (0)	13.2
GICOR-GOECP-SEOR Study (2021)^[3]^	Phase 3 RCT	NCF in HA-PCI vs cPCI	150	HA-PCI: 17 (22.6) cPCI: 13 (17.3)	HA-PCI: 3 (4.0)	40.4
NCT01780675 study (2021)^[4]^	Phase 3 RCT	NCF in HA-PCI vs cPCI	168	HA-PCI: 14 (16.7) cPCI: 17 (20.2)	HA-PCI: 5 (6.0)	26.6
Cho et al (2021)^[12]^	Single center	HA-PCI vs cPCI	106	HA-PCI: 10 (20.8) cPCI: 11 (18.7)	HA-PCI: 2 (4.2)	21

cPCI = conventional prophylactic cranial irradiation; HA = hippocampal-avoidance; NCF = neurocognitive function; RCT = randomized controlled trial.

While HA-WBRT is recognized as a viable treatment option for SCLC patients, perihippocampal failure may be attributable to the under-dosing of radiation during planning coverage or to the aggressiveness of the tumor itself. The optimal management of SCLC patients with recurrent brain metastasis has also changed significantly in recent years. Treatment options include salvage systemic therapy or stereotactic radiosurgery.^[8]^ The prognosis for SCLC patients with systemic or brain failure is poor, but advances in targeted therapy and immunotherapy may improve outcomes.^[19]^ More research into this topic is encouraged.

## Acknowledgments

The authors would like to thank Enago^®^ for the English language review.

## Author contributions

**Data curation:** Yi-Chia Ho, Li-Tsun Shieh, Chia-Hui Lin, Chia-Chun Chen.

**Writing – original draft:** Yi-Chia Ho, Sheng-Yow Ho.

**Conceptualization:** Sheng-Yow Ho.

**Funding acquisition:** Sheng-Yow Ho.

**Investigation:** Sheng-Yow Ho.

**Supervision:** Sheng-Yow Ho.

**Validation:** Sheng-Yow Ho.

**Writing – review & editing:** Sheng-Yow Ho.
